# From Ancient Fruit to Functional Innovation: Liposomal Delivery of Haritaki (*Terminalia chebula*) Fruit Extract via Chocolate Matrix

**DOI:** 10.3390/antiox15030362

**Published:** 2026-03-12

**Authors:** Danijela Šeremet, Predrag Petrović, Iva Budimir, Petra Vukosav, Tea Mišić Radić, Ana Butorac, Aleksandra Vojvodić Cebin, Rada Pjanović, Svjetlana Škrabal, Draženka Komes

**Affiliations:** 1 Department of Food Engineering, Faculty of Food Technology and Biotechnology, University of Zagreb, Pierottijeva 6, 10000 Zagreb, Croatia; danijela.seremet@pbf.unizg.hr (D.Š.); budimiriva99@gmail.com (I.B.); aleksandra.vojvodic@pbf.unizg.hr (A.V.C.); 2 Innovation Centre, Faculty of Technology and Metallurgy, University of Belgrade, Karnegijeva 4, 11000 Belgrade, Serbia; ppetrovic@tmf.bg.ac.rs; 3 Division for Marine and Environmental Research, Ruđer Bošković Institute, Bijenička 54, 10000 Zagreb, Croatia; petra.vukosav@irb.hr (P.V.); tmisic@irb.hr (T.M.R.); 4 Selvita Ltd., Prilaz Baruna Filipovića 29, 1000 Zagreb, Croatia; ana.butorac@selvita.com; 5 Department of Chemical Engineering, Faculty of Technology and Metallurgy, University of Belgrade, Karnegijeva 4, 11000 Belgrade, Serbia; rada@tmf.bg.ac.rs; 6 Faculty of Tourism and Rural Development in Požega, Josip Juraj Strossmayer University of Osijek, Vukovarska 17, 34000 Požega, Croatia; sskrabal@ftrr.hr

**Keywords:** chocolate, encapsulation, haritaki, liposomes, polyphenols

## Abstract

In the present study, the fruit extract of haritaki (*T. chebula*) was successfully encapsulated in liposomes, achieving an encapsulation efficiency of 97.2% for the total polyphenols, with the most significant release occurring under simulated digestive conditions in the intestinal phase. The zeta potential and Z-average size of the loaded liposomes were 20.52 mV and 521.73 nm at a pH of 2 and −59.72 mV and 823.03 nm at a pH of 8, respectively. The prepared liposomes were further incorporated in the matrix of dark chocolate in a content of 10%. The addition of liposomes significantly (*p* < 0.05) increased the particle size distribution (d(0.9), d(0.5) and d(0.1)) and the rheological (Casson’s yield point and viscosity) parameters of the chocolate, while the hardness and maximum melting temperature did not change significantly (*p* > 0.05). The results of the sensory analysis of the chocolates confirmed that the liposomes were well homogenized in the chocolate matrix and that the herbal taste of haritaki was successfully masked by incorporating it into the chocolate in the encapsulated form.

## 1. Introduction

Extracts from different fruits possess various health benefits, such as anti-inflammatory, antioxidant, antitumor and antidiabetic activity, etc. These biological activities are due to the presence of biologically active compounds—vitamins, polyphenols, carotenoids, polysaccharides, dietary fiber, alkaloids, essential oils, etc.—which have been shown to be more beneficial when present together [1]. In the food industry, fruit extracts can be used as sweeteners, as they naturally contain sugars; as natural flavoring and coloring agents; and as ingredients in the development of functional foods [1,2]. The fruit extract market size was valued at USD 15.21 billion in 2022 and is expected to grow from USD 15.98 billion in 2023 to USD 25.0 billion in 2032, at a CAGR of 5.1% [3].

*Terminalia* is a genus comprising mostly deciduous trees, many of which have nutritious and edible fruits with medicinal value. *Terminalia chebula*, known as haritaki or harada, is one of them and is called the “king of medicine” in Tibet due to its healing power [4]. In the area of Asia and Europe, haritaki fruit is used as a dietary supplement, while in South Asia, it is commonly consumed as a sweet dish called Harad ka murabba (jam), prepared by soaking the berries in sugar syrup [5]. Haritaki fruits are rich in polyphenolic compounds and vitamin C [6] and *in vivo* studies have shown their hepatoprotective [7], antidiabetic [8], antihyperlipidemic [9] and neuroprotective [10] activities, as well as many other properties. A particularly interesting study is that of Chakkalakal et al. [11], which showed that oral supplementation with a haritaki fruit extract with highly enriched hydrolysable tannins can reduce sebum excretion and improve facial appearance parameters, such as erythema and facial wrinkle severity. It is worth noting that haritaki does not appear to have any debilitating or toxic side effects [6]. Encapsulation is very often used to improve the bioavailability, and thus the biological activity of polyphenols and other bioactive compounds; it is a technique in which the core material is enclosed within a wall material, resulting in capsules that offer protection against chemical and environmental factors [12]. Jha and Sit [13] formulated freeze-dried microparticles containing encapsulated *T. chebula* extract with potato starch as the carrier material and obtained high encapsulation efficiency, while Senthivel et al. [14] successfully produced polymeric nanoparticles loaded with *T. chebula* extract using hydroxypropyl methyl cellulose and a solvent evaporation method. Jha and Sit [15] even formulated functional yogurt fortified with free and encapsulated freeze-dried *T. chebula* extract. Spray-drying and freeze-drying are still the most used encapsulation techniques [16], but liposomal encapsulation is also being increasingly investigated. Since liposomes are characterized by a high biocompatibility and biodegradability, as well as a low potential for triggering immune reactions, they have become the most used nanocarriers for a range of hydrophobic and hydrophilic bioactive compounds. Structurally, liposomes are spherical colloidal particles that form spontaneously in solution through the organization of amphiphilic molecules such as phospholipids [17]. Their membranes can consist of one or more lipid bilayers (lamellae) surrounding an inner aqueous core, with the hydrophilic head groups orientated towards both the inner and outer aqueous phases [18]. The encapsulation of bioactive compounds from haritaki in liposomes has not yet been scientifically investigated, but a similar study was conducted by Varma et al. [19], who formulated phytosomes of haritaki extract. The key distinction between phytosomes and liposomes lies in the location of the active compound—in liposomes, it is either dispersed within the internal cavity or embedded in the membrane layers, whereas in phytosomes, the active compound forms a stable part of the membrane itself by binding via hydrogen bonds to the polar head groups of phospholipids [20].

The global food encapsulation market was estimated to reach USD 7.1 billion in 2025 and is projected to reach USD 18.0 billion by 2035, at a CAGR of 9.8% in the period of 2025–2035. Consumer demand for functional and clean label ingredients is a factor driving industry growth, and companies are expanding their product portfolio with the help of advanced encapsulation technologies [21]. Therefore, in line with the current market demands, the aim of this study was to formulate liposomes loaded with haritaki extract in order to increase its bioavailability and, further, to use in the formulation of innovative chocolates. The formulated liposomal delivery system with encapsulated haritaki extract was subjected to the characterization of bioactive (encapsulation efficiency and *in vitro* digestion), physico-chemical (Z-average size, zeta potential and FT-IR spectroscopy) and morphological (AFM microscopy) properties, while newly formulated chocolate with the incorporated liposomes was characterized by determination of the particle size distribution and the bioactive, rheological, textural, melting and sensory properties.

## 2. Materials and Methods

### 2.1. Materials

The solvents, reagents, enzymes and analytical standards were supplied by Merck (Darmstadt, Germany), Carlo Erba (Cornaredo, Italy)), Fisher Scientific (Hampton, NH, USA), Gram mol d.o.o. (Zagreb, Croatia), Kemika d.d. (Zagreb, Croatia) and Lach-Ner (Neratovice, Czech Republic). Phospholipon^®^ 90G was purchased from Lipoid GmbH (Ludwigshafen, Germany), cocoa mass and cocoa butter by Barry Callebaut (Zürich, Switzerland), powdered sugar by Franck d.d. (Zagreb, Croatia) and sunflower lecithin by Nutrimedica d.o.o. (Zagreb, Croatia). Synastol^®^ TC (Sytheon, Boonton, NJ, USA) was used as the *Terminalia chebula* fruit extract.

### 2.2. Methods

#### 2.2.1. Liposomal Encapsulation of the Haritaki Extract

The liposomal encapsulation of the haritaki extract was carried out according to the procedure described by Perrett et al. [22]. Phospholipon^®^ 90 G (1 g) was used for the liposome preparation, and it was dissolved in the same amount of ethanol (1 g) in a laboratory flask with stirring (2 min) and heating to 60 °C. After cooling, the haritaki extract (100 mg), previously dissolved in a 70% methanolic solution (1 mL), was added to the same flask with the dissolved phospholipid. A total of 50 mL of demineralized water was added to the resulting solution with constant stirring using a peristaltic pump at a flow rate of 15 mL/h. Plain liposomes were also prepared in the described manner, but without the addition of the extract.

#### 2.2.2. Determination of Encapsulation Efficiency

An amount of 1 mL of the prepared liposome suspension was added to an Eppi^®^ tube and centrifuged (SL8/8R, Thermo Scientific, Waltham, MA, USA; 9500 rpm, 45 min, 4 °C). The supernatant was then separated into a separate tube for analysis and the remaining liposome residue was washed with 3 mL of demineralized water and centrifuged again (9500 rpm, 45 min, 4 °C). The supernatant was discarded and the remaining liposome residue was suspended in 1 mL of demineralized water and transferred to a screw-cap tube. A total of 1 mL of methanol and 1 mL of chloroform were added to the same tube and the contents of the tube were vortexed (1 min). The phase separation was accelerated by brief centrifugation. Afterwards, the upper aqueous methanolic phase containing encapsulated polyphenols was transferred to a tube and used for analysis. The encapsulation efficiency was defined by determining the content of the total and individual polyphenols as the ratio of their mass concentration determined in the separated aqueous methanolic phase to the sum of the same concentration and the concentration determined in the first separated supernatant.

Content of total phenolic compounds was determined using the spectrophotometric method of Singleton and Rossi [23]. Content of individual phenolic compounds was determined by HPLC-DAD methodology on an Agilent Series 1200 chromatographic system (Agilent Technologies, Santa Clara, CA, USA), following the method of Šeremet et al. [24]. Using this HPLC-DAD methodology, gallic and ellagic acids were identified and quantified. Identification was carried out by comparing the retention times and characteristic absorption spectrums (190–400 nm) with available standards and quantification by establishing calibration curves (20–100 μg/mL). The obtained chromatograms also revealed other peaks and to identify those, the haritaki extract was subjected to fractionation using an Agilent 1260 Infinity II Analytical-Scale Fraction Collector (Agilent Technologies, Santa Clara, CA, USA) coupled with the previously mentioned Agilent Series 1200 chromatographic system. Peak-based collected fractions were analyzed by a TripleQuad LC/MS system with an electrospray ion source (Agilent Technologies, Santa Clara, CA, USA) in the positive and negative MS scan mode. Ion source parameters were set at a gas temperature of 250 °C, a gas flow of 7 L/min, a nebulizer at 40 psi, a sheath gas heater set to 325 °C, sheath gas flow to 11 L/min, a capillary of voltage 3500 V and a fragmentor voltage of 200 V. A multiple-reaction-monitoring method was developed using the precursor ions and major fragment ions described in the study by Pfundstein et al. [25]. Identification of polyphenolic compounds was conducted by comparing the obtained m/z (mass-to-charge ratio) to the ones published in the same study [25]. In this way, punicalin(4,6-O-(S,S)-gallagyl-α/β-D-Glc), 1,6-Di-O-galloyl-2,4-chebuloyl-β-D-Glc(or 1,3-), chebulinic acid(1,3,6-tri-O-galloyl-2,4-O-chebuloyl-β-D-Glc) and 1,2,3,4,6-Penta-O-galloyl-β-D-Glc were identified. Their quantification was conducted by the previously mentioned HPLC-DAD methodology and using gallic acid as a standard (20–100 μg/mL).

#### 2.2.3. *In Vitro* Digestion of Liposomes

The release kinetics of haritaki polyphenols from liposomes were monitored using simulated gastric fluid (SGF) and simulated intestinal fluid (SIF) solutions prepared according to the method of Minekus et al. [26]. In addition to the appropriate inorganic salts, the SGF solution contained pepsin (2000 U/mL in the solution) and the pH was adjusted to 3.0 by adding hydrochloric acid. The SIF solution contained appropriate inorganic salts, as well as pancreatin (1 mg/mL) and bile salts (2 mg/mL), and the pH was adjusted to 7.0 by adding sodium hydroxide. The liposomes were transferred to 30 mL of the SGF solution at 37 °C with constant stirring on a magnetic stirrer. At appropriate time intervals, aliquots of 100 µL were taken from the solution until the last sample was taken after 2 h. The SGF solution with liposomes was then transferred to 30 mL of SIF solution under the same conditions. The procedure for taking aliquots at specific time intervals was repeated as previously described. The aliquots were used to determine the content of total polyphenols [23], which was expressed as the mg gallic acid equivalents (GAE) per gram of liposomes or extract. For samples collected during the SGF phase, the blank contained an equal volume of SGF instead of the sample, and for samples collected during the SIF phase, it contained a mixture of SGF and SIF in the same ratio (*v*/*v*, 1:1). *In vitro* digestion of the haritaki extract (in free form) was also performed in the same way as described for liposomes.

#### 2.2.4. Physical Characterization of Liposomes

Z-average size and zeta potential of plain and loaded liposomes were determined on a Malvern Nano-ZS Zetasizer (Malvern, UK) in the pH range from 2 to 8.

#### 2.2.5. Morphological Characterization of Liposomes by AFM

AFM (atomic force microscopy) imaging of liposomes was performed using a multimode scanning probe microscope equipped with a NanoScope IIIa controller (Bruker, Billerica, MA, USA) and a scanner with a 125 nm lateral engagement (JV). The experiments were conducted in tapping mode with silicon probes (TESP V2, nominal resonance frequency of 320 kHz, nominal spring constant of 42 N/m, Bruker). The set point to free amplitude ratio (A/A_0_) was maintained at 0.9 to ensure light tapping and reduce tip–sample interaction forces. The linear scan rate was optimized between 1.0 and 1.5 Hz, with an imaging resolution of 512 samples per line. Image processing and analyseswere performed using the NanoScope Analysis software (Bruker, version 3.0). Except for first-order two-dimensional flattening, the images are shown as raw data. All measurements were conducted in air at room temperature. For AFM sample preparation, the liposome suspension was diluted to 1:40 in ultrapure water. A 5 μL aliquot of the diluted sample was deposited onto freshly cleaved mica. The mica substrates were then placed in sealed Petri dishes for 30 min to allow the solvent to evaporate, after which AFM imaging was performed.

#### 2.2.6. Fourier-Transform Infrared (FT-IR) Spectroscopy

The FT-IR spectra of the haritaki extract and the plain and loaded liposomes were obtained using the attenuated total reflectance (ATR) mode in the range of 550–4000/cm on aNicolet iS10 (Thermo Scientific, Waltham, MA, USA) spectrometer. Prior to the analysis, all samples were prepared in freeze-dried form.

#### 2.2.7. Formulation of Chocolates

The chocolate samples were prepared by mixing all ingredients in a melanger (Twin Stone, Hollywood, FL, USA) for 2 h. Cocoa liquor and cocoa butter were preheated to 50 °C and added to the melanger in liquid form, followed by the addition of powdered sugar and sunflower lecithin. Two formulations were prepared: the control sample (C_CON), which contained no liposomes and consisted of cocoa mass (75%), cocoa butter (9%), sugar (15%) and lecithin (1%), and the second formulation (C_LIP), which contained 10% liposomes loaded with the haritaki extract, which was added after the sugar and before the lecithin. After mixing, the chocolate mass was tempered manually—first heated to 45–50 °C, then cooled to 28 °C, finally heated to 32 °C with constant stirring and then poured into molds. The molded chocolates were stored at +4 °C for 8 h before demolding.

#### 2.2.8. Bioactive Characterization of Chocolates

To characterize the bioactive composition of the chocolates, the total phenolic content [23] and the antioxidant capacity were determined by the DPPH [27] and ABTS [28] methods. For that purpose, samples of the prepared chocolates (3 g) were melted in a water bath (Inko VKZ ERN, Inkolab d.o.o., Zagreb, Croatia) and then 10 mL of boiling water was added. Extraction was carried out for 15 min on a magnetic stirrer (SMHS-6, Witeg Labortechnik GmbH, Wertheim, Germany) and then also for 15 min in an ultrasonic bath (Elmasonic S 60 H, Elma, Germany; nominal power of 200 W, frequency of 37 kHz) heated at 50 °C. The samples were then cooled and 10 mL of methanol was added. The extraction was carried out again for 15 min on a magnetic stirrer and then for 15 min in an ultrasonic bath (50 °C). The samples were then centrifuged (9500 rpm, 20 min) and the collected supernatants were used for the analysis of the bioactive composition (total phenolic content and antioxidant capacity). The release kinetics of polyphenols from chocolates under simulated digestion conditions were determined as described in Section 2.2.3.

#### 2.2.9. Rheological Characterization of Chocolates

The rheological properties of the chocolate samples were evaluated according to the IOCCC method [29] using a modular compact rheometer, MCR102 (Anton Paar GmbH, Graz, Austria), operated with the RheoCompass 1.3. software (Anton Paar, Austria). The Casson model was used to determine the yield stress and viscosity of the samples.

#### 2.2.10. Determination of Particle Size Distribution of Chocolates

The particle size distribution of the formulated chocolates was determined on a Malvern Mastersizer 2000 particle size analyzer with the 2000S Hydro liquid sampler (Malvern, UK). The device was connected to a computer and controlled by the Mastersizer 2000 5.60 program. The chocolates (10 g) were homogenized in sunflower oil (300 mL) before measurement. The device was calibrated before starting the measurement process. The sample was then gradually added, with the degree of saturation displayed on the computer screen. The particle size distribution parameters analyzed for these samples were d(0.9), d(0.5) and d(0.1).

#### 2.2.11. Textural Characterization of Chocolates

The chocolate samples were subjected to a hardness analysis, expressed as the penetration force, using the TA.HD.plus texture analyzer(Stable Micro Systems, Godalming, UK). Dimensions of the samples were 3 cm × 2.5 cm × 1 cm (length × width × height), and they were tempered to room temperature before the analysis. To maximize the compression-to-shear ratio, a flat-bottomed cylindrical steel probe, P/2 (Stable Micro Systems, Godalming, UK), with a diameter of 2 mm, was used to pierce the samples. The penetration depth was set to 10 mm with a penetration speed of 0.5 mm/s. The resulting diagrams were processed using the Texture Exponent 6.1.8.0. software (Stable Micro Systems, Godalming, UK).

#### 2.2.12. Determination of Melting Properties of Chocolates

Differential scanning calorimetry (DSC) was performed using a Mettler Toledo DSC 823e device according to the method described in the study by Dolatowska-Żebrowska et al. [30]. Each sample (about 4 mg) was enclosed in a dedicated aluminum measuring cup. The samples were analyzed in the device by cooling to 10 °C and then heating at a rate of 4 °C/min in the temperature range from 10 °C to 50 °C with an inert nitrogen stream at a flow rate of 50 mL/min. The values for the maximum melting temperature (*T_m_*) and the melting enthalpy (*ΔH_m_*) for cocoa butter of form V (β_2_) in the chocolate samples were read from the obtained DSC thermograms.

#### 2.2.13. Sensory Analysis of Chocolates

The sensory evaluation was carried out in accordance with the ISO 8589:2007 standard [31], by a trained internal panel consisting of 10 members (aged 20–50) from the University of Zagreb Faculty of Food Technology and Biotechnology. Before the evaluation, the chocolate samples were brought to room temperature. The evaluation criteria included visual characteristics (color, gloss and surface appearance), auditory perception during breaking, and textural characteristics in terms of the melting behaviour and taste attributes (sweetness, bitterness and herbal notes). Each sensory attribute was rated on a 9-point intensity scale, with 1 being the lowest and 9 being the highest perceived intensity. The overall product acceptability was rated on a 9-point hedonic scale, ranging from 1 (extremely dislike) to 9 (extremely like). A more detailed description of the selection of the panel members, as well as a description of their training and the method for conducting the sensory analysis, is described in the work by Šeremet et al. [32].

#### 2.2.14. Statistical Analysis

The data are given as mean values with standard deviations. The statistical analysis was performed with the Statistica software (v.14, TIBCO Software Inc., Palo Alto, CA, USA) using Student’s *t*-test. Differences were classified as statistically significant at *p* < 0.05.

## 3. Results and Discussion

### 3.1. Bioactive Characterization of Liposomes

**Figure 1:**
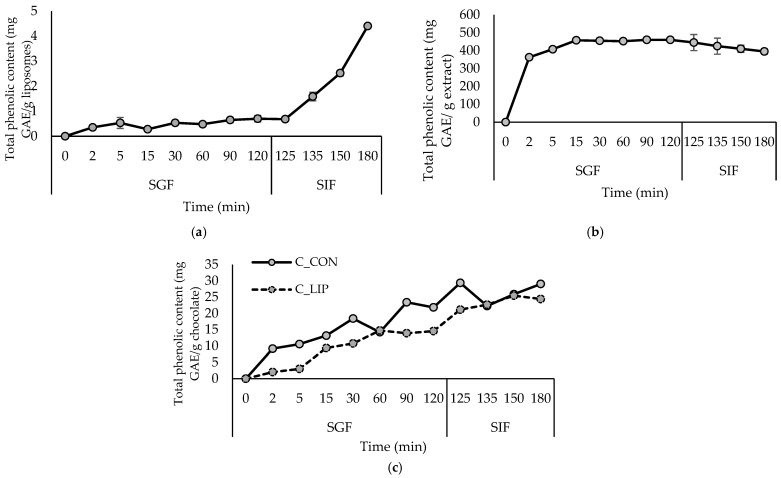
Release kinetics of total polyphenols from liposomes with encapsulated haritaki extract (**a**), haritaki extract in free form (**b**) and formulated chocolates (**c**) in simulated digestion in the stomach (SGF) and small intestine (SIF). GAE—gallic acid equivalents.

The bioactive characterization of the loaded liposomes was performed by determining the encapsulation efficiency (EE) of haritaki polyphenols and their release under simulated digestion in the stomach and small intestine. The results are shown in Table 1 and Figure 1a.

The encapsulation of haritaki polyphenols in liposomes was successful, since a high encapsulation efficiency (EE) for the total polyphenols (97.2%) was obtained. Regarding the individual polyphenols, the EE of gallic acid was 62.9%, and for the gallate ester—1,2,3,4,6-Penta-O-galloyl-β-D-Glc, it was 99.1%. From the group of chebulic ellagitannis, 1,6-Di-O-galloyl-2,4-chebuloyl-β-D-Glc(or 1,3-) and chebulinic acid(1,3,6-tri-O-galloyl-2,4-O-chebuloyl-β-D-Glc) were identified and their EEs were 97.6 and 92.4%, respectively. The EE for ellagic acid was also high—more precisely 97.2%. Considering the hydrophilic nature of gallic and ellagic acids, simple gallate esters and chebulic elagitannins, it is very likely that they are located in the hydrophilic core of the liposomes and on the liposome surface as well (Section 3.4). Varma et al. [19] reported an entrapment efficiency of 77.56% for the haritaki extract in phytosomes formulated using a ratio of phospholipid and cholesterol of 1.5:1.2, an extract concentration of 1% and an alcohol concentration of 50%.

According to the results of the simulated gastrointestinal digestion (Figure 1a), the release of polyphenols from liposomes in the gastric phase (SGF) was extremely slow, which is to be expected, since pepsin in the SGF is inactive towards phospholipids, so they cannot be hydrolyzed in the stomach [33]. During the intestinal phase, the release of polyphenols accelerated due to the presence of bile salts and pancreatin in the SIF, which affected the physical properties and morphology of the liposomes. Pancreatic lipase contained in pancreatic enzymes catalyzes the hydrolysis of phospholipids, and the detergent properties of bile salts lead to disruption of the phospholipid bilayers in liposomes [33,34]. Given that the small intestine plays a key role in the absorption of nutritive and bioactive compounds, such a release profile is expected to improve the bioavailability of haritaki polyphenols [35]. The protective and controlled-release effect of liposomes in the gastric phase is evident when comparing the release kinetics to those of the haritaki extract in free form (Figure 1b). During *in vitro* digestion, the extract in free form showed a rapid release of polyphenols in the gastric phase, followed by a relatively stable or slightly decreasing content during the intestinal phase.

### 3.2. Physical Characterization of Liposomes

**Figure 2:**
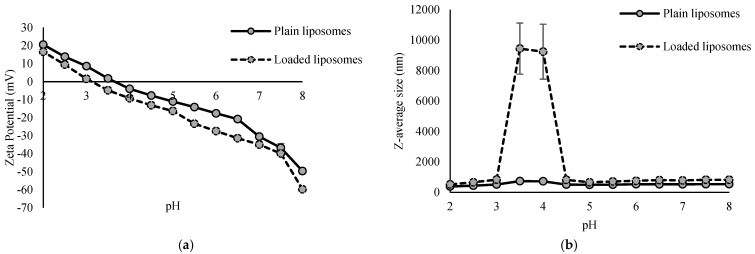
Zeta potential (**a**) and Z-average size (**b**) of plain and loaded liposomes at different pH values.

The physical characterization of the plain and loaded liposomes included determination of the zeta potential and Z-average size for a pH range from 2 to 8. The results are presented in Figure 2.

Zeta potential of both plain and loaded liposomes decreased with increasing pH values (Figure 2a). The zeta potential represents the total surface charge of particles and is influenced by factors such as the lipid composition, the type of lipid headgroups, the attached ligands, the environment and its ionic strength. It can vary from negative to neutral or positive [18]. Zeta potential of plain liposomes at a pH of 2 was 20.52 mV, and at a pH of 8, it was −49.63 mV. Similar values were observed with loaded liposomes—the zeta potential at a pH of 2 was 20.52 mV, and at a pH of 8, it was −59.72 mV. Isoelectric point of plain liposomes was 3.41, and for loaded liposomes, it was 3.82. Throughout the whole pH range, loaded liposomes had a lower value for the zeta potential than plain liposomes, indicating the adsorption and/or incorporation of the extract compounds on the surface, whose deprotonated functional groups contributed to the additional negative charge. This pH-dependent change indicates complex intermolecular interactions between the extract and the lipid bilayer membrane of liposomes.

The size of liposomes is determined by the number of lamellae (lipid bilayers), and it ranges from 20 nm to even more than 1000 nm [18]. Z-average size of plain liposomes was in the range from 380.50 nm, at a pH value of 2, to 737.73 nm, at a pH value of 3.5. Loaded liposomes showed relative stability in their Z-average size over the whole pH range, except from 3.5 to 4, where values of the Z-average size were even up to 9241 nm and 9444 nm, respectively, which is the area of their isoelectric point. Outside this range, the size values were from 521.73 nm (pH of 2) to 830.83 nm (pH of 3) (Figure 2b). In the case of plain liposomes, the increase in size at the isoelectric point was not so pronounced. This sudden increase in size most likely indicates aggregation or destabilization of loaded liposomes in that pH range. The zeta potential at the isoelectric point is 0 mV, meaning that the electrostatic repulsion between particles is minimal, resulting in aggregation of liposomes and thus explaining the strong increase in the Z-average size (Figure 2b). Under these conditions, electrostatic repulsion no longer exists and van der Waals forces prevail, resulting in aggregation. Liposome aggregation was not irreversible, since upon a further pH increase, the system restabilized, as evidenced by a reduction in the Z-average size and a return to the initial or even lower values. Further, in the present study, both plain and loaded liposomes over the whole pH range had a Z-average size greater than 100 nm, indicating the presence of at least one double lipid bilayer [36]. Varma et al. [19] reported size of phytosomes with encapsulated haritaki extract in the range from 226.32 nm to 338.89 nm, depending on the formulation, i.e., ratio of phospholipids and cholesterol, and the extract and alcohol concentration in the phytosomes.

The liposomes were prepared for incorporation into dark chocolate, which exhibited a pH value of approximately 6. Given that zeta potential values greater than +30 mV or less than −30 mV indicate a good liposomal stability against coalescence [37], it can be concluded that the liposomes are likely to remain relatively stable within the chocolate matrix.

### 3.3. Morphological Characterization of Liposomes

**Figure 3:**
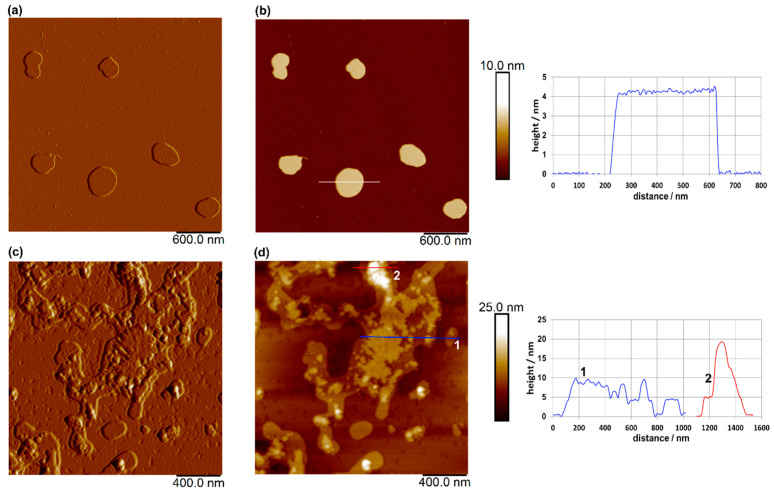
AFM images of plain liposomes on a mica surface: amplitude image (**a**) and topographic image (**b**), and of loaded liposomes on a mica surface: amplitude image (**c**) and topographic image (**d**). Vertical profiles along the indicated lines in (**b**,**d**) are shown next to the corresponding images. Images were acquired in tapping mode in air, with scan sizes of 3 μm × 3 μm for (**a**,**b**), and 2 μm × 2 μm for (**c**,**d**), and vertical scales of 10 nm for (**b**) and 25 nm for (**d**).

The results of AFM imaging of plain and loaded liposomes are shown in Figure 3.

AFM imaging of the plain liposomes revealed well-defined, spherical-like vesicles with lateral dimensions of 250–410 nm, consistent with the Z-average size measurements (Figure 2b), and an average height of approximately 4.3 nm. Compared to plain liposomes, the loaded liposomes exhibited a rougher and more heterogeneous surface morphology, with less-uniform vesicle boundaries and irregular topographic features (Figure 3c,d). These changes may indicate interactions between the incorporated extract and the lipid bilayer, which can alter the structural organization of the membrane. Despite the observed aggregation, evidenced also by the determination of the Z-average size (Figure 2b) and morphological irregularities, vesicle-like structures were still clearly distinguishable in the AFM images, suggesting that the overall liposomal structure was largely preserved after loading. The presence of these structures, rather than amorphous deposits, further indicates that the extract was associated with the liposomal system rather than existing solely as a separate phase.

Šeremet et al. [32] reported similar heights (5 nm) for plain liposomes and those loaded with ground ivy extract, which were prepared using Phospholipon^®^ 90G, as well as widths ranging from 60 to 460 nm. The authors [32] also revealed that the encapsulation of ground ivy extract could potentially disrupt the spherical shape of the liposomes. Batinić et al. [38] also reported comparable liposome height values (4–12 nm) for formulations prepared with Phospholipon^®^ 90G, cholesterol and a surfactant for folic acid encapsulation. The authors [38] observed a flattened and partially collapsed morphology following the incorporation of folic acid into the liposomal structure.

### 3.4. FT-IR Spectroscopy

**Figure 4:**
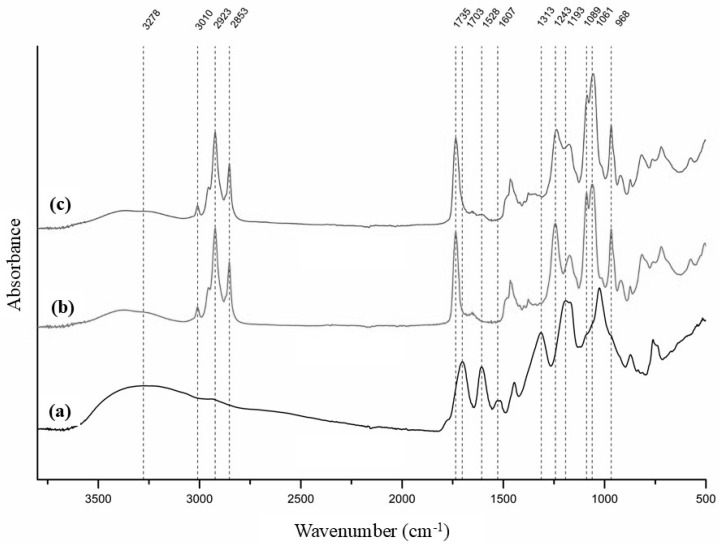
FT-IR spectra of haritaki extract (**a**), plain liposomes (**b**) and loaded liposomes (**c**).

The FT-IR spectra of the haritaki extract and the plain and loaded liposomes are presented in Figure 4.

In the FT-IR spectrum of the haritaki extract, a broad absorption band centered at 3278 cm^−1^ corresponded to O–H stretching vibrations. Bands at 1703 and 1607 cm^−1^ were attributed to carboxyl groups, representing undissociated and dissociated forms in equilibrium. The doublet at 1528 and 1516 cm^−1^ arose from C=C stretching vibrations, while the peak at 1313 cm^−1^ may be related to O–H bending of both alcohol and phenol groups, indicating a high content of these functional groups. A cluster of bands in the 1200–800 cm^−1^ region was assigned to various C–O, C–O–C and C–C vibrations typically present in alcohols, carboxylic acids and sugars.

The FT-IR spectrum of plain liposomes exhibited characteristic absorption bands confirming the presence of phosphatidylcholine. An absorption band observed at approximately 3010 cm^−1^ was assigned to the stretching vibrations of olefinic (=C–H) groups originating from unsaturated fatty acid chains. Prominent bands at 2923 and 2853 cm^−1^ were attributed to the asymmetric and symmetric stretching vibrations of CH_2_ groups from fatty acid chains. The absorption band at 1735 cm^−1^ was ascribed to C=O stretching vibrations of the ester linkage between glycerol and fatty acids. The asymmetric and symmetric stretching vibrations of phosphate (PO_2_^−^) groups were observed at 1243 and 1089 cm^−1^, respectively, while the band at 1061 cm^−1^ was assigned to C–O stretching vibrations. Additionally, the quaternary ammonium group of the choline headgroup gave rise to characteristic bands at 968 and 920 cm^−1^, attributed to vibrational modes associated with the N^+^(CH_3_)_3_ moiety [39,40].

Despite performing the FT-IR analysis immediately after freeze-drying, the spectrum revealed O–H stretching in the 3600–3200 cm^−1^ region, indicating the presence of hydrogen-bonded water. This was likely due to water adsorbed onto the bilayer surface, particularly interacting with phosphate groups via hydrogen bonding.

The FT-IR spectrum of the loaded liposomes was similar to that of the plain liposomes, but due to the interaction of the extract compounds with the phosphate groups of the liposome, a slight shift in the phosphate (PO_2_^−^) groups, stretching bands from 1243 cm^−1^ to 1237 cm^−1^ and from 1089 to 1086 cm^−1^ was observed. This suggests that at least part of the extract polyphenols are in the hydrophilic core and on the surface of the liposomes, where the phosphate groups are situated [41]. Further evidence that the extract compounds may be located on the surface of the liposomes is the presence of a band at 1607 cm^−1^ attributed to carboxyl groups, which could also explain why the loaded liposomes had a lower zeta potential than the plain liposomes (Section 3.2).

### 3.5. Bioactive Characterization of Chocolate Samples

The bioactive composition, including the total phenolic content and antioxidant capacity, of the chocolate samples is presented in Table 2.

The control chocolate sample (C_CON), prepared without the addition of liposomes, contained 22.95 mg GAE/g of total polyphenols and consequently possessed a high antioxidant capacity (0.10 and 0.11 mmol Trolox/g). The control sample contained 84% cocoa solids, which explains its rich bioactive composition. In the sample prepared with the addition of liposomes (C_LIP), a significantly lower (*p* < 0.05) content of total polyphenols, 17.38 mg GAE/g, was determined. Although sample C_LIP contained a lower content of total polyphenols than C_CON, their values of antioxidant capacities were not statistically significantly (*p* > 0.05) different, indicating a strong antioxidant capacity of the polyphenols in the haritaki extract.

Monitoring the kinetics of the polyphenols released under simulated digestion was performed to verify the possibility of a continuous and controlled release of the encapsulated polyphenolic compounds, even after incorporation into the food product. The results are shown in Figure 1c. In the chocolate sample with the addition of liposomes (C_LIP), a continuous release of polyphenols was observed during simulated digestion in the stomach (SGF) and small intestine (SIF), indicating that the encapsulated extract was gradually released from the liposomal carriers within the chocolate matrix. In the control chocolate sample (C_CON), polyphenols were also released during digestion, although to a lesser extent, reflecting the natural release of cocoa-derived phenolic compounds from the chocolate matrix. Chocolates are usually enriched with polyphenolic compounds by incorporating various plant extracts. For example, in the study by Šeremet et al. [32], the successful incorporation of liposomes loaded with ground ivy extract into chocolate was also visible in achieving a controlled and continuous release of polyphenolic compounds under conditions of simulated digestion in the stomach and small intestine. Gültekin-Özgüven et al. [42] formulated dark chocolate fortified with spray-dried black mulberry waste extract encapsulated in chitosan-coated liposomes. The highest anthocyanin content (31.1 mg cyanidin-3-glucoside/L) was determined in chocolate prepared by adding the liposomes in natural (pH of 4.5) cocoa liquor during the last hour of conching at a temperature of 40 °C, while the control sample, prepared under the same conditions, had 21.0 mg cyanidin-3-glucoside/L of anthocyanin content.

### 3.6. Particle Size Distribution of Chocolate Samples

**Figure 5:**
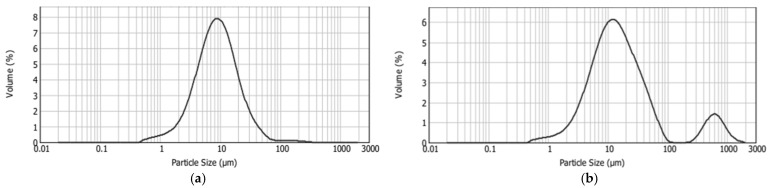
Size distribution of control chocolate (C_CON) (**a**) and chocolate with liposomes (C_LIP) (**b**).

The results of analyzing the particle size distribution of the chocolate samples, expressed as d(0.1), d(0.5) and d(0.9), are shown in Table 2, and the particle size distribution curves are shown in Figure 5.

From the obtained results, it is evident that the incorporation of liposomes into the chocolate matrix caused a statistically significant (*p* < 0.05) increase in all the particle size distribution parameters. The range of the optimal particle size in chocolate is generally considered to be between 17 and 30 µm, while particles exceeding 30 µm tend to produce a gritty texture in the mouth [43]. In the control sample (C_CON), 90% of the particles were smaller than 23.90 µm, which is within the range of optimal smoothness in the mouth, while in the chocolate sample with liposomes (C_LIP), 90% of the particles were smaller than 74.15 µm. However, this should not immediately imply a grainy mouthfeel, as the d(0.5) parameter for sample C_LIP was 14.01 µm, i.e., 50% of the particles were smaller than this size.

The particle size distribution curve of sample C_CON was unimodal, i.e., it contained one peak (Figure 5a), whereas the curve of sample C_LIP was bimodal, i.e., it contained two peaks (Figure 5b), indicating the heterogeneity of the system. The presence of a second peak in sample C_LIP was due to the presence of larger particles, which may have resulted from liposome agglomerates or interactions of the liposomes with other chocolate components.

### 3.7. Rheological Properties of Chocolate Samples

**Figure 6:**
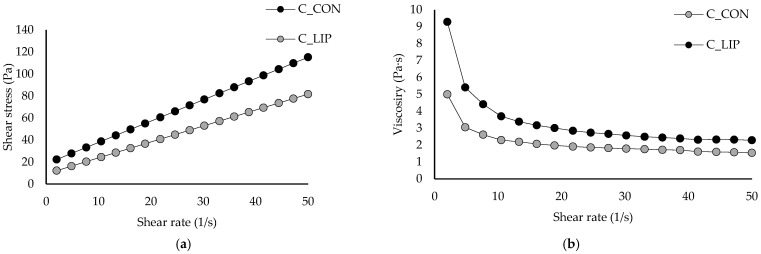
Flow curves (**a**) and viscosity curves (**b**) of prepared chocolates.

Based on the flow (Figure 6a) and viscosity (Figure 6b) curves, all chocolate samples showed pseudoplastic behaviour characterized by increased shear stress and decreased viscosity with increasing shear rate. The rheological parameters were evaluated using the Casson model and the corresponding results are summarized in Table 2.

The value of the Casson yield stress for the control sample (C_CON) was 2.78 Pa and it was statistically significantly (*p* < 0.05) increased to 7.21 Pa by the addition of liposomes into the chocolate matrix (sample C_LIP). The higher value of Casson yield stress in the sample C_LIP could indicate stronger interactions between the ingredients than in the sample C_CON. According to the obtained results, the sample C_LIP therefore requires a higher shear stress value (7.21 Pa) above which it behaves like a liquid compared to the control sample C_CON (2.78 Pa), i.e., it behaves like a solid at a higher shear stress than the control sample C_CON. The Casson viscosity for the control sample C_CON was 1.04 Pa·s and was also statistically significantly (*p* < 0.05) increased to 1.25 Pa·s by the addition of liposomes (sample C_LIP). Liposomes are spherical nanoparticles that can act as solid particles in suspension, and an increased number of these particles can enhance interparticle interactions (among liposomes, cocoa solids and sugar) and thus result in a higher viscosity.

Similar conclusions were reported by Hadnađev et al. [44], who fortified chocolates with protein (soy, whey and potato)-stabilized spray-dried fish oil microcapsules in contents of 1.97% and 3.94%. The authors concluded that the increase in plastic viscosity and yield stress after the addition of microcapsules could be attributed to a higher content of solid particles, without a corresponding increase in the fat phase. Samples containing microcapsules were characterized by a lower amount of the freely movable fat phase and more pronounced particle–particle interactions, which can be explained by a reduced amount of free fat phase trapped within the crystalline network between the particles. As a result, this hindered the flow of the tested samples, leading to an increase in viscosity. On the contrary, Didar [45] reported that the Casson viscosity of dark chocolate was not significantly affected by the incorporation of liposomal vitamin D3 in a content of 0.5 µg/g (1.26 and 1.25 Pa·s).

### 3.8. Textural Properties of Chocolate Samples

The hardness, as one of the most important textural characteristics of the formulated chocolates, was measured, and the results are shown in Table 2.

The hardness of the control sample (C_CON) was 30.86 N and that of the sample with the addition of liposomes (sample C_LIP) was 30.39 N. According to the obtained results, the addition of liposomes into the chocolate matrix had no statistically significant (*p* > 0.05) effect on the hardness of the chocolate. It can be concluded that liposomes can be added to increase the functionality of chocolate without negatively affecting its texture and without forming pores that would soften the chocolate.

Muhammad et al. [46] observed that the incorporation of nanoparticles (0.5, 1, 1.5 and 2%) made of shellac, xanthan gum and cinnamon extract into milk and white chocolate led to an increase in hardness. They explained that the addition of nanoparticles also increased the moisture content, which could influence the formation of sugar networks and strengthen the aggregated particle-to-particle network, thereby increasing the breaking strength of the chocolate. In addition, the authors hypothesized that the nanoparticles increased the solid volume fraction in the chocolate, leading to more particle interactions and, ultimately, a firmer texture. In a study by Šeremet et al. [32] it was shown that the addition of 4% liposomal ground ivy extract to chocolate only led to a reduction in hardness if the liposomes had previously been homogenized in cocoa butter. Conversely, no such effect was observed when the liposomes were mixed directly into the chocolate mass. The authors hypothesized that homogenization in cocoa butter could disrupt the liposomal bilayer, possibly causing the phosphatidylcholine to act as an emulsifier. In contrast, no reduction in hardness was observed in the present study, which could mean that the integrity of the bilayer of liposomes containing the haritaki extract remained intact during incorporation into the chocolate matrix.

### 3.9. Melting Properties of Chocolate Samples

**Figure 7:**
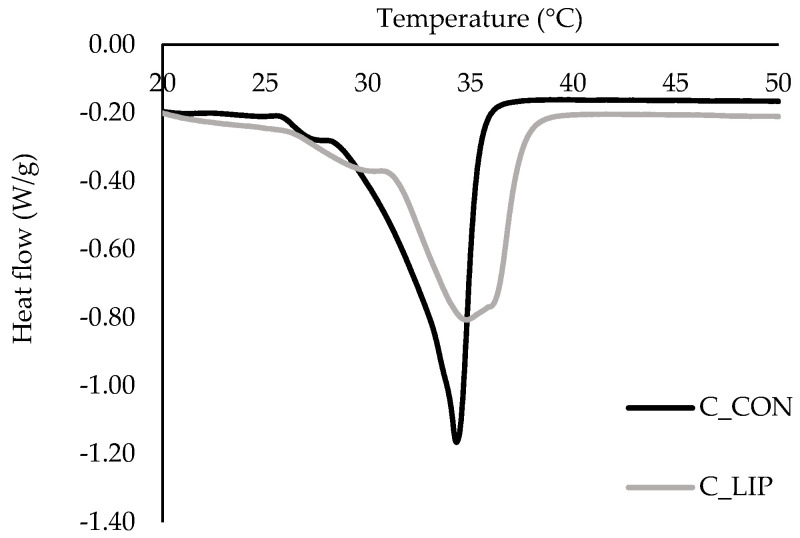
DSC thermograms of prepared chocolates.

Thermograms of the chocolate samples in the form of DSC curves are shown in Figure 7, and the melting parameters are shown in Table 2.

Two endothermic peaks were observed in both chocolate samples. The first peak was related to the presence of form IV (β_1_) of cocoa butter, which should melt in the temperature range of 26 to 28 °C [47]. The second endothermic peak in both samples was in the temperature range of ~ 30–38 °C, indicating the presence of forms V (β_2_) and VI (β_1_) of cocoa butter. The control sample (C_CON) had a pronounced and sharper peak with an onset temperature of 28.2 °C, a maximum melting temperature of 34.3 °C and an end temperature of 36.1 °C, indicating the dominance of form V of cocoa butter [48]. Sample C_LIP had a broader and less intense peak with an onset temperature of 30.8 °C, a maximum melting temperature of 34.8 °C and an end temperature of 37.9 °C, indicating a mixture of forms V and VI of cocoa butter [48]. Regardless of that, maximum melting temperatures (34.3 and 34.8 °C) of the samples did not differ significantly (*p* > 0.05). It is possible that the addition of liposomes caused a slower and more controlled cooling of the chocolate, promoting the formation of more stable crystal forms because the lipid molecules had sufficient time to crystallize into more thermodynamically favorable forms. It is known that the addition of specific phospholipids to chocolate can affect the crystallization of cocoa butter. Stobbs et al. [49] reported that 1,2-dimyristoyl-sn-glycero-3-phosphocholine (DMPC) directs the crystallization of cocoa butter to the desirable form V of cocoa butter, through forming micelles in butter, which then serve as a seeding surface, templating form V crystal growth via its effects on POS (palmitoyl-oleoyl-stearoyl glycerol).

Further, the values for melting enthalpy (*ΔH_m_*) of the samples were statistically significantly (*p* < 0.05) different, and it was found that the amount of energy required to melt the control sample C_CON was 47.36 J/g and the amount of energy required to melt the sample C_LIP was 34.8 J/g. Although a more stable crystalline form VI of cocoa butter was detected in the chocolate sample with liposomes (C_LIP), the melting enthalpy was lower than in the control sample (C_CON), which contained only form V of cocoa butter. This result can be explained by the potential dilution of the crystalline phase in sample C_LIP due to the presence of liposomes and the possible disruption of the ordered formation of the cocoa butter crystal network. Liposomes can also accelerate the transition from form V to form VI of cocoa butter, but such a transition may not lead to a higher thermal melting energy if the structure is inhomogeneous or the content of form VI is relatively low. Therefore, the statistically significant (*p* < 0.05) decrease in *ΔH_m_* for sample C_LIP compared to sample C_CON may be attributed to a combination of changes in the crystal structure, dilution of the fat phase and possible interactions of the liposomes with the chocolate matrix.

Didar [45] reported that addition of liposomal vitamin D3 (0.5 µg/g) in dark chocolate did not affect the peak temperature and energy required for the complete melting of the sample, as determined by a DSC analysis. On the contrary, Šeremet et al. [32] reported that the addition of liposomal encapsulates of ground ivy extract in chocolate in a content of 4% led to a reduction in the maximum melting temperature, as the free fat phase in the chocolate matrix was reduced by coating the liposomes with cocoa butter.

### 3.10. Sensory Evaluation of Chocolate Samples

**Figure 8:**
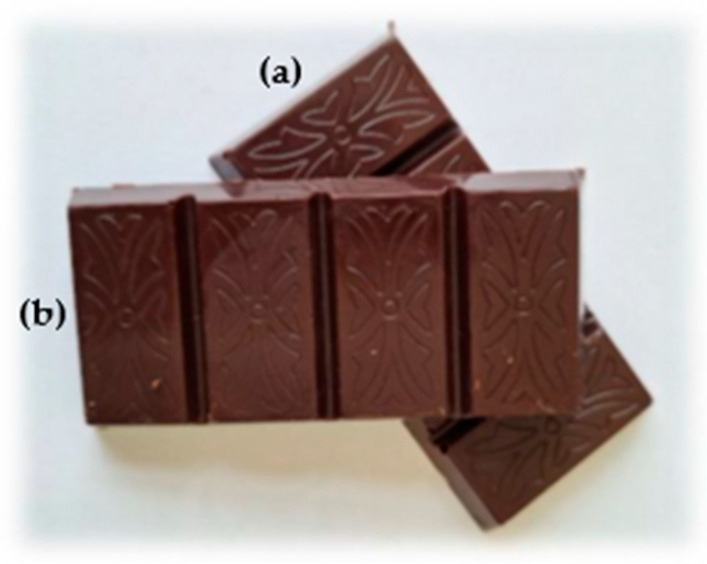
Formulated chocolate samples—C_CON (**a**) and C_LIP (**b**).

**Figure 9:**
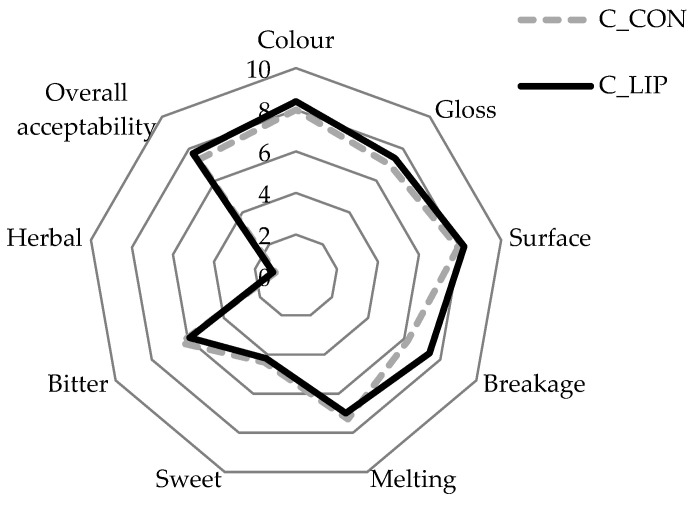
Sensory analysis of prepared chocolates.

The formulated chocolates are shown in Figure 8 and were sensory evaluated for their appearance (color, gloss and surface), acoustics (breakage), texture (melting), taste (sweet, bitter and herbal) and overall acceptability. The results are presented in Figure 9.

Both chocolate samples were rated highly in terms of their appearance parameters. The color score for the control sample (C_CON) was 8.0 and for the chocolate prepared with the addition of liposomes (C_LIP), it was 8.4. The gloss and surface of the chocolates were also rated with similar scores—sample C_CON with 7.0 and 7.9, respectively, and sample C_LIP with 7.4 and 8.2, respectively. The high scores for the visual appearance of sample C_LIP indicate a good homogenization of the liposomes into chocolate matrix. The breakage intensity was higher for sample C_CON (7.4) than for sample C_LIP (6.2). As breakage intensity is very often associated with hardness, it is interesting to note that, despite the different scores for breakage intensity, the hardness of the chocolate samples was not statistically significantly (*p* > 0.05) different (Section 3.8). Further, the melting intensity was rated similarly for both chocolate samples (7.3 and 7.0), indicating that the addition of the liposomes did not affect the melting profile of the chocolate. The same was found by the DSC analysis—the maximum melting temperature (peak temperature) of the samples did not differ significantly (*p* > 0.05) (Section 3.9). The chocolates were evaluated as medium sweet (4.4 and 4.2), as expected due to the low sugar content in the formulation, while the bitter taste was more pronounced (6.4 and 5.9), which was also expected given the high cocoa content in the formulation. The herbal taste of the haritaki extract was successfully masked by the encapsulation, but also by the incorporation into the chocolate, as the herbal taste was rated with very low scores in both chocolate samples (1.0 and 1.1). Finally, both chocolates were well accepted by the panel members, with the sample C_LIP receiving a higher overall acceptance rating (7.7) than the control sample C_CON (7.3).

## 4. Conclusions

Liposomes proved to be efficient delivery systems for the haritaki polyphenols in terms of a high encapsulation efficiency and pronounced release in the intestinal phase during simulated digestion. The high antioxidant capacity of dark chocolate was maintained by the incorporation of liposomes, as well as the ability of liposomes to ensure a controlled and continuous release of the encapsulated polyphenols when incorporated into the chocolate matrix. The interaction between liposomes and chocolate compounds was detectable by an increase in the Casson yield stress and viscosity, a change in the particle size distribution parameters (bimodal size distribution curve) and a decrease in the melting enthalpy. Although the change in the particle size distribution curve and the decrease in the melting enthalpy could indicate an inhomogeneous system of chocolate with liposomes, inhomogeneity was not observed in the sensory analysis. The chocolate with liposomes was evaluated with high scores for visual appearance and received a similar rating from the sensory panel as the chocolate control for melting intensity. The maximum melting temperature of the chocolate, as determined by DSC, also did not change with the incorporation of liposomes, nor did the hardness, so it can be concluded that liposomes with encapsulated haritaki extract can be used to formulate innovative chocolates without changing their features that are most important to consumers.

## Figures and Tables

**Table 1:** Liposomal encapsulation efficiency of total and individual polyphenols.

Encapsulation Efficiency (%)						
Total polyphenols	Gallic acid	1,2,3,4,6-Penta-*O*-galloyl-β-D-Glc	Punicalin(4,6-O-(*S*,*S*)-gallagyl-α/β-D-Glc)	1,6-Di-*O*-galloyl-2,4-chebuloyl-β-D-Glc(or 1,3-)	Chebulinic acid(1,3,6-tri-*O*-galloyl-2,4-*O*-chebuloyl-β-D-Glc)	Ellagic acid
97.2 ± 1.7	62.9 ± 2.1	99.1 ± 0.4	98.2 ± 1.1	97.6 ± 1.4	92.4 ± 0.9	92.7 ± 2.8

**Table 2:** Bioactive and physical characterization of chocolates.

Sample	Bioactive Composition			Size Distribution			Rheological Properties		Textural Properties	Melting Properties	
	Total Polyphenols (mg GAE/g)	Antioxidant Capacity (mmol Trolox/g)		d(0.1)	d(0.5)	d(0.9)	Casson Yield Stress (Pa)	Casson Viscosity (Pa·s)	Hardness (N)	Maximum Melting Temperature (°C)	Enthalpy of Melting (J/g)
		DPPH	ABTS								
C_CON	22.95 ± 0.33 *	0.10 ± 0.01	0.11 ± 0.01	3.04 ± 0.13 *	8.75 ± 0.07 *	23.90 ± 1.03 *	2.78 ± 0.06 *	1.04 ± 0.02 *	30.86 ± 2.70	34.3 ± 0.1	47.4 ± 1.3 *
C_LIP	17.38 ± 0.08 *	0.11 ± 0.01	0.11 ± 0.01	4.04 ± 0.08 *	14.01 ± 0.18 *	74.15 ± 1.62 *	7.21 ± 0.09 *	1.25 ± 0.02 *	30.39 ± 0.34	34.8 ± 0.0	34.8 ± 0.7 *

GAE—gallic acid equivalents; *—statistically significant difference (*p* < 0.05) between samples determined by Student’s *t*-test.

## Data Availability

The original contributions presented in this study are included in the article. Further inquiries can be directed to the corresponding author.

## Abbreviations

The following abbreviations are used in this manuscript:

AFM	Atomic Force Microscopy
FT-IR	Fourier-Transform Infrared
SGF	Simulated Gastric Fluid
SIF	Simulated Intestinal Fluid
DSC	Differential Scanning Calorimetry
